# Bioinformatory‐assisted analysis of next‐generation sequencing data for precision medicine in pancreatic cancer

**DOI:** 10.1002/1878-0261.12108

**Published:** 2017-08-08

**Authors:** Linnéa Malgerud, Johan Lindberg, Valtteri Wirta, Maria Gustafsson‐Liljefors, Masoud Karimi, Carlos Fernández Moro, Katrin Stecker, Alexander Picker, Carolin Huelsewig, Martin Stein, Regina Bohnert, Marco Del Chiaro, Stephan L. Haas, Rainer L. Heuchel, Johan Permert, Markus J. Maeurer, Stephan Brock, Caroline S. Verbeke, Lars Engstrand, David B. Jackson, Henrik Grönberg, Johannes‐Matthias Löhr

**Affiliations:** ^1^ Center for Digestive Diseases Karolinska University Hospital Stockholm Sweden; ^2^ Department of Clinical Sciences Intervention and Technology (CLINTEC) Karolinska Institutet Stockholm Sweden; ^3^ Department of Medical Epidemiology & Biostatistics (MEB) Karolinska Institutet Stockholm Sweden; ^4^ Science for Life Laboratory Department of Microbiology, Tumor and Cell Biology (MTC) Karolinska Institutet Stockholm Sweden; ^5^ Department of Oncology at Radiumhemmet Karolinska University Hospital Stockholm Sweden; ^6^ Department of Pathology Karolinska University Hospital Stockholm Sweden; ^7^ Molecular Health GmbH Heidelberg Germany; ^8^ Innovation Office Karolinska University Hospital Stockholm Sweden; ^9^ Department of Laboratory Medicine (LABMED) Karolinska Institutet Stockholm Sweden

**Keywords:** bioinformatics, drug–drug interactions, evidence‐based, NGS, pancreatic cancer

## Abstract

Pancreatic ductal adenocarcinoma (PDAC) is a tumor with an extremely poor prognosis, predominantly as a result of chemotherapy resistance and numerous somatic mutations. Consequently, PDAC is a prime candidate for the use of sequencing to identify causative mutations, facilitating subsequent administration of targeted therapy. In a feasibility study, we retrospectively assessed the therapeutic recommendations of a novel, evidence‐based software that analyzes next‐generation sequencing (NGS) data using a large panel of pharmacogenomic biomarkers for efficacy and toxicity. Tissue from 14 patients with PDAC was sequenced using NGS with a 620 gene panel. FASTQ files were fed into treatmentmap. The results were compared with chemotherapy in the patients, including all side effects. No changes in therapy were made. Known driver mutations for PDAC were confirmed (e.g. *KRAS*,*TP53*). Software analysis revealed positive biomarkers for predicted effective and ineffective treatments in all patients. At least one biomarker associated with increased toxicity could be detected in all patients. Patients had been receiving one of the currently approved chemotherapy agents. In two patients, toxicity could have been correctly predicted by the software analysis. The results suggest that NGS, in combination with an evidence‐based software, could be conducted within a 2‐week period, thus being feasible for clinical routine. Therapy recommendations were principally off‐label use. Based on the predominant *KRAS* mutations, other drugs were predicted to be ineffective. The pharmacogenomic biomarkers indicative of increased toxicity could be retrospectively linked to reported negative side effects in the respective patients. Finally, the occurrence of somatic and germline mutations in cancer syndrome‐associated genes is noteworthy, despite a high frequency of these particular variants in the background population. These results suggest software‐analysis of NGS data provides evidence‐based information on effective, ineffective and toxic drugs, potentially forming the basis for precision cancer medicine in PDAC.

**Abbreviations**
5‐FU: 5‐fluorouracil
ATM: ATM serine/threonine kinase
BRCA1: breast cancer 1
CDA: cytidine deaminase
DPYD: dihydropyrimidine dehydrogenase
DRDB: Drug Response Database
FDA: Food and Drug Administration
FFPE: formalin‐fixed paraffin‐embedded
FL‐Oxa: fluorouracil leucovorin oxaliplatin
FOLFIRINOX: fluorouraci leucovorin irinotecan and oxaliplatin
KRAS: Kirsten rat sarcoma viral oncogene
MLH1: MutL homolog 1
MSH: MutS protein homolog 2
NGS: next‐generation sequencing
PDAC: pancreatic ductal adenocarcinoma
RTK: receptor tyrosine kinase
TP53: tumor protein 53

## 1 Introduction

Pancreatic ductal adenocarcinoma (PDAC) is the fourth leading cause of cancer‐related mortality in the USA and Europe (Siegel *et al*., 2014) and is predicted to become the second by 2030 (Rahib *et al*., 2014). Death from pancreatic cancer now excedes breast cancer in Europe (Ferlay *et al*., 2016). Unlike breast or colorectal cancer, pancreatic cancer is always terminal (Löhr, 2006). At diagnosis, approximately 80–90% of pancreatic cancer patients are inoperable with therapy‐resistant locally advanced or metastatic disease. The median survival is approximately 6 months (Bond‐Smith *et al*., 2012). Even with the best available therapeutic regimens, median survival time does not exceed 10 months (Conroy *et al*., 2011a,b; Von Hoff *et al*., 2013). The 5‐year survival rate for all stages of pancreatic cancer has remained close to 5% for the past 25 years and is the lowest for any cancer despite numerous efforts to improve the treatment for PDAC patients (Bond‐Smith *et al*., 2012; Michl and Gress, 2013; Sohal *et al*., 2014). PDAC poses one of the greatest unmet medical needs in cancer research and can be regarded as a medical emergency (Löhr, 2014). The lack of treatment response to conventional therapeutic approaches as radiation and chemotherapy is attributable to many factors, including extrinsic or intrinsic resistance (Michl and Gress, 2012; Wang *et al*., 2011).

Pancreatic cancers may benefit from the developments in precision medicine, which has proven worthy elsewhere. This has been a result of the identification of discriminating tumor markers and the development of targeted therapeutic options, a prime example being hormone receptors and receptor tyrosine‐protein kinase erbB‐2 expression in breast cancer, as well as proto‐oncogene c‐Kit in gastrointestinal stromal tumors. Although these therapies target single biomarkers and PDAC has a heterogeneous mutational landscape, the identification of single biomarkers is a first step in personalized drug combination therapy (Kris *et al*., 2014). Approximately 5–10% of PDAC patients respond to targeted therapy against vascular endothelial growth factor or rapidly accelerated fibrosarcoma/rat sarcoma viral oncogene homolog kinase (Garrido‐Laguna and Hidalgo, 2015); however, we lack the tools to identify them (Garrido‐Laguna *et al*., 2015). Because of this dire situation, sequencing has specifically been proposed in pancreatic cancer, allowing mutational cancer analysis to become a prognostic and diagnostic tool readily available to clinicians (Mardis, 2012).

As a result of extensive sequencing efforts, such as the Human Genome Project and The Cancer Genome Atlas, it is becoming clear that identifying singular abnormalities (e.g. mutations in the Kirsten rat sarcoma viral oncogene [*KRAS]* oncogene or the tumor protein 53 [TP53] tumor suppressor gene from sequencing data) is not sufficient to make therapeutic decisions (Martincorena *et al*., 2015). Several retrospective studies nevertheless demonstrate convincing explanations for treatment response, or failure, depending on the collective genetic make‐up of the tumor (Gentzler *et al*., 2014; Kim *et al*., 2014), which highlights an emerging interest in more complex and sophisticated software tools and algorithms.

The adaptation of next‐generation sequencing (NGS) assays to formalin‐fixed, paraffin‐embedded tissue (FFPE) (Frampton *et al*., 2013; Holley *et al*., 2012) and even fine‐needle biopsy material (Young *et al*., 2013) in pancreatic cancer patients has further facilitated the potential integration of NGS data into clinical practice. To date, only a few studies have prospectively used a sequencing approach and based treatment decisions on the genetic information obtained. These demonstrate a clear survival benefit for the personalized therapy based on pharmacogenomic biomarkers over conventional standard‐of‐care therapy (Kris *et al*., 2014; Tsimberidou *et al*., 2014), thus creating a discussion amongst the stakeholders on how to conduct and finance these studies, as well as on how to reimburse personalized cancer medicine in the future (Lewis *et al*., 2013).

It is therefore necessary to use an integrated analysis of the sequencing data from a given tumor that takes into account the entire body of knowledge on that particular tumor entity and all of the information available for possible treatment options, including their side effects and interactions with other drugs. Furthermore, given the vast amount of data generated, it is clear that the information handed over to the treating physician cannot be raw bioinformatics data and should be presented in an interpreted, clinically relevant and user friendly format (Ellard *et al*., 2013; Gullapalli *et al*., 2012). treatmentmap(Molecular Health, Heidelberg, Germany) is such an evidence‐based system for data analysis that provides physicians with tumor profiles based on genome‐sequencing data from a single patient, as well as an objective list of all available scientific and medical data supporting the decision.

In the present study, we aimed to investigate the clinical applicability of using NGS in combination with the software tool (treatmentmap) to generate individualized analysis for a personalized approach for the treatment of pancreatic cancer. We report a feasibility study demonstrating the successful implementation of NGS with treatmentmap into the clinical workflow with the initial results providing a rationale for future studies.

## 2 Materials and methods

### 2.1 Patients and study set‐up

This was an open prospective feasibility study aiming to establish NGS in the clinical setting within the framework of our patient‐driven process at the Center for Digestive Diseases, Karolinska University Hospital, with patient recruitment between March 2014 and December 2014. The study was approved by the local ethics committee (EPN; Diarie‐Nr. 2013/2:10). Patients with pancreatic adenocarcinoma who were willing to join the study were provided with information and required to provide informed consent. The tumor material was collected during surgical resection of the tumor, although there was an additional patient included who was not resectable where the tissue was collected from a liver metastases. As a control, either adjacent nontumor tissue (duodenal or gastric) or an EDTA blood sample that was collected at the time of surgery was used.

### 2.2 DNA extraction and sequencing

Existing hemotoxylin and eosin slides were reviewed by the expert pathologist (CV), assuring the correct histological diagnosis of ductal adenocarcinoma of the pancreas. A block was selected for DNA extraction with a tumor content of at least 20% in line with the prerequisites for NGS and use of the software. DNA extraction was performed with standard protocols using the QIAmp DNA tissue kits (fresh frozen and FFPE tissue samples, as well as blood). DNA was fragmented using Covaris S2 sonicator (Covaris, Woburn, MA, USA) to an average of 100 bp (FFPE tissues) and 300 bp (blood) depending on DNA quality. DNA target enrichment was performed manually using optimized protocols (e.g. prolonged hybridization times, optimized PCR cycles and washing steps) for SureSelectXTall exon V5 Plus (Agilent Technologies Inc., Santa Clara, CA, USA) for whole exome and custom for the SeqCap EZ (NimbleGen, Waldkraiburg, Germany) custom 620 gene panel under study (Table S1). DNA quality control was performed with Life Technologies Qubit Fluorometer and Agilent Bioanalyzer 2100 or an AATI fragment Analyzer at several steps throughout the process. Sequencing was performed using a HiSeq 2500 (Illumina, San Diego, CA, USA) (rapid‐run mode with paired‐end 2 × 100 bp reads) FASTQ generation and demultiplexing was performed using Casava, version 1.8.4 (Illumina). The average fragment length was 200–400 bp. The average coverage achieved was > 100 ×.

### 2.3 Data processing and software algorithm

An evidence‐based expert system for data analysis was used (treatmentmap). As input information, treatmentmap processes genome‐sequencing data from a single patient, together with basic clinical and demographic patient parameters. This information is then analyzed in three major steps: (i) genome analysis; (ii) evidence mining; and (iii) clinical interpretation, which are further described here.

#### 2.3.1 Step 1: genome analysis

The first major step is the genome data analysis. Here, the system detects genetic alterations in a patient's tumor, based on an analysis of their raw sequencing data. Targeted panel sequencing information is analyzed in a nonpaired fashion and does not include a comparison with the patient's germline reference. The genome analysis pipeline uses a defined set of quality‐controlled, standard analytical applications and reference resource databases that are connected in a controlled workflow. The tools of the pipeline were selected by evaluating sensitivity and precision using synthetic patient data with know variants (R. Bohnert, S. Vivas, & G. Jansen, re‐submitted).

In terms of detailed steps, the genome analysis pipeline takes raw sequence data as input (FASTQ format), together with associated clinical data (i.e. patient diagnosis, age, sex, ethnicity). The genome analysis pipeline has to align the sequence data with the ancestry specific reference genomes. The generated BAM (binary alignment map) files are then processed through the respective algorithm for variant calling, which can detect gene fusion, indels and single nucleotide variants. Tumor‐ and germline‐specific genomic alterations are then mapped to unique reference proteins using Ensembl DB homo_sapiens_core (http://www.ensembl.org) and UniProt (http://www.uniprot.org). The system determines the longest best protein isoform as reference sequence for mapping to the information in the proprietary Nucleus knowledgebase that is part of the software.

#### 2.3.2 Step 2: evidence mining

Once the tumor has been analyzed, the next step of the treatmentmap analytical workflow is to automatically identify all previously published knowledge about the clinical implications of genetic alterations. Accordingly, the treatmentmap system screens all genotype information against the reference information on genes, pathways, biological pathways, variants, treatments, clinical trials, etc., in the Nucleus knowledgebase. In the core of this information is a manually curated database of biomarker information: the so‐called Drug Response Database (DRDB). To aid this quality assured process, the biomedical curation team uses text data mining algorithms and manually classifies pharmacogenomic biomarkers according to three levels of clinical validity (Table 1).

**Table 1:** Levels for grading evidence based on treatmentmap.

Quality level 1: Clinically endorsed pharmacogenetic FDA‐approved biomarkers: Highest relevance information
Quality level 2: Clinically observed biomarkers (i.e. observations stemming from clinical data but not yet FDA‐approved: High relevance information
Quality level 3: Translational level biomarkers characterized in preclinical studies and/or predicted by bioinformatics algorithms: Information of low or unclear relevance

Such quality and relevance measures are not only important to this analysis, but also are reported directly in the treatmentmap report, ensuring that they are explicitly clear about how clinically actionable a pharmacogenomic biomarker finding might be for their patient. Other essential information captured during the curation process is also included: (i) The variant (i.e. the type of genomic aberration: SNP, Insertion or Deletion etc.); (ii) the drug or treatment used; (iii) the effect of the variant on treatment (i.e. response, resistance or toxicity); (iv) the quantity of effect (e.g. strong, medium, weak); (v) the observation context (i.e. the disease/disease stage or model system); and (vi) a link to the source information and a grading of its reliability.

The DRDB database includes information about any form of genomic aberration including single nucleotide variants, copy number variations, fusion proteins, insertions and deletions, and combinations thereof. The lineage of the mutation is also captured; for example, whether it is a germline or somatic mutation. Similarly, the database includes information about the drug or treatment associated with a pharmacogenomic (i.e. genomic aberration) being reported, as well as the source of the information (e.g. seen in model systems or patients), and includes MeSH terms (Medical Subject Headings) and other hierarchical classifications. Variants were matched against mutations logged in the Human Gene Mutation Database (HGMD^®^Professional) (http://www.biobase-international.com/hgmd) from BIOBASE Corporation (http://www.hgmd.org) (Stenson *et al*., 2009).

The information contained within the DRDB patient and/or tumor mutation profile serves to determine a patient's likelihood of response to therapy, likelihood of resistance to therapy and likelihood of toxicity.

#### 2.3.3 Step 3: clinical interpretation

treatmentmap provides analytical results and access to biomedical resources for a reliable evidence‐based clinical interpretation of the genetic alterations via a web‐based user interface. This online report displays the genetic alterations detected in the tumor genome and the potential effects of these alterations on (i) drug efficacy (i.e. whether the detected genotype confers likelihood of response or resistance to cancer drug treatments) and (ii) drug toxicity (i.e. increased likelihood that the patient might experience adverse drug effects). In addition to the established pharmacogenomic biomarker information, further metrics are provided, such as automated assessments of the importance of a gene in a particular cancer type using a new method referred to as oncoscoring, in addition to a prediction of the functional impact of the aberration on gene/protein function, referred to as ‘functional impact scoring’. Known public functional impact scoring tools (https://omictools.com/functional-predictions-category) are commonly prediction tools using machine‐learning approaches. By contrast, the functional impact scoring system that is implemented in treatmentmap is basing on an evidence‐associated weighted sum of features scoring.

The oncoscore method is a programmed tool that relies on multidimensional data types summarizing real‐time evidence about clinical and molecular importance with respect to specific cancer indications. Features include: gene/protein pathway inclusion facts, drug targets, disease association and interaction neighborhood, as well as indication‐specific protein and targetable attributes. Applying these parameters across individual cancer indications allows a prioritization of the functionally most important genes associated with each cancer type. To understand the impact of aberrations, we contextualized structural, functional, drug response and safety information to provide a novel approach to the prediction of functionally important aberrations. The oncoscore serves to rationally prioritize genes and their specific variants with respect to the disease.

PharmGKB, another drug–drug interaction and pharmacogenetic database based on the Food and Drug Administration (FDA) adverse event reporting system was used for cross‐reference and validation (Thorn *et al*., 2010; Whirl‐Carrillo *et al*., 2012). PharmGKB's prediction of the drugs most likely to cause adverse drug reactions to the patients was compared with the treatmentmap data.

### 2.4 Follow‐up

Patients were followed according to clinical routines. Previous therapy, side effects from chemotherapy and second line therapies were recorded.

## 3 Results

The 14 patients received several chemotherapy regimens, in some cases sequential: gemcitabine monotherapy (*n* = 8); fluorouracil leucovorin oxaliplatin (FL‐Oxa) (*n* = 3); gemcitabine + abraxane (*n* = 1); gemcitabine + capcitabine (*n* = 1); fluorouracil leucovorin irinotecan and oxaliplatin (FOLFIRINOX) (*n* = 2) (Table 2). The disease duration (i.e. from the time when surgery was performed until the genetic analysis) was 0.5–24 months (median 12 months). The median survival time after the date of diagnosis was 19.5 months, with three patients still alive at the time of data closure.

For all 14 tumor samples analyzed, the median of target coverage ranged from 123 to 212. For the control samples, the median of target coverage ranged from 41 to 223. The tumor samples showed a ≥ 100 × depth in 71.1–94.2% of targeted sites and the control samples showed a ≥ 100 × depth in 2.5–81.9% of targeted sites. The turnaround time for NGS was 10 days with respect to software analysis and reporting took 2 days on average.

The somatic genetic changes identified in these 14 patients are well in line with known driver mutations in pancreatic cancer (Kamisawa *et al*., 2016) (Table 3): 13 had *KRAS* mutations (9 × G12D), four patients had additional *TP53* mutations, 10 had additional *EGFR* (including one with wild‐type *TP53*) and three had additional SMAD4 (mothers against decapentaplegic homolog 4) mutations. In a subset of patients (*n* = 10) where sufficient quality sequencing data were available, analysis of germline and somatic mutations in the gene panel could be performed (Table S2). Besides the germ line mutations in drug metabolizing enzymes (e.g. dihydropyrimidine dehydrogenase; DPYD), a number of germ line variants could be found in breast cancer 1 (BRCA1) (*n* = 6), MutS protein homolog 2 (MSH) 2/6 (*n* = 5), ATM serine/threonine kinase (ATM) (*n* = 4), MutL homolog 1 (MLH1) (*n* = 4) and mismatch repair endonuclease PMS2 (PMS2) (*n* = 4) (Tables S2 and S3); however, these particular variants have a high prevalence in the background population and are presumably without clinical significance. There was no record of a positive family history for pancreatic cancer in these patients (Table S2).

**Table 2:** Clinicopathogical characteristics and molecular profile of the 14 patients

Sample ID	Sex	Age	TNM stage	Resection	Systemic treatments	Survival time	Recommended treatments	Treatments to avoid	Increased risk of toxicity
EUP100	F	63	T3N1M0	Total pancreatectomy, splenectomy, vascular resection (SMV + CHA), R1	Gemcitabine FL‐Oxa	18 months, 17 days	Trametinib + Docetaxel AKT inhibitor MEK + PK inhibitor IGF‐1R antibody + Temsirolimus	Single agent RTK‐inhibitors Everolimus	Cisplatin Cyclophosphamide
EUP101	M	70	T3N1M0	Whipple, R1	Gemcitabine FL‐Oxa	10 months, 17 days	Trametinib + Docetaxel AKT inhibitor MEK + PK inhibitor	Single agent RTK‐inhibitors Everolimus Imatinib	Capecitabine Cisplatin Cyclophosphamide
EUP102	F	65	T3N0M0	Whipple, R1	Gemcitabine	36 months, 2 days^a^	Trametinib + Docetaxel AKT inhibitor MEK + PK inhibitor	Single agent RTK‐inhibitors Everolimus	Trastuzumab Capecitabine Cisplatin Cyclophosphamide Paclitaxel
EUP103	F	67	T3N1M0	Whipple, vascular resection (PV), R1	Gemcitabine Irinotecan + capecitabine	10 months, 22 days	Trametinib + Docetaxel AKT inhibitor MEK + PK inhibitor	Single agent RTK‐inhibitors Imatinib Everolimus	Paclitaxel Trastuzumab Capecitabine
EUP104	F	81	T3N1M0	Whipple, R1	None	14 months, 23 days	Trametinib AKT inhibitor MEK + PK inhibitor IGF‐1R antibody + Temsirolimus	Single agent RTK‐inhibitors Everolimus	Cisplatin Mercaptopurine Thioguanine Trastuzumab
EUP105	M	65	T3N1M0	Whipple, R0	None	16 months, 1 day	Trametinib AKT inhibitor MEK + PK inhibitor IGF‐1R antibody + Temsirolimus	Single agent RTK‐inhibitors Everolimus	Capecitabine Cisplatin Cyclophosphamide
EUP106	M	59	T3N1M1	Whipple, R0	Gemcitabine	6 months, 18 days	Paclitaxel Trametinib + Docetaxel AKT inhibitor MEK + PK inhibitor IGF‐1R antibody + Temsirolimus Epirubicin	Single agent RTK‐ inhibitors Everolimus Gefitinib	Capecitabine Cisplatin Cyclophosphamide
EUP107	K	66	T3N1M0	Total pancreatectomy, R1	Gemcitabine	15 months, 23 days	Trametinib + Docetaxel AKT inhibitor MEK + PK inhibitor	Single agent RTK‐inhibitors Everolimus	Capecitabine
EUP108	M	50	T3N1M0	Whipple, R1	Gemcitabine FL‐Oxa	9 months, 1 day	Trametinib + Docetaxel AKT inhibitor MEK + PK inhibitor	Single agent RTK‐inhibitors Everolimus	Capecitabine
EUP109	F	75	T3N1M0	Whipple, R1	None	6 months, 7 days	Trametinib + Docetaxel	Single agent RTK‐inhibitors Everolimus	Cisplatin Cyclophosphamide
EUP110	M	62	T3N1M0	Whipple, R1	Gemcitabine GemCap	32 months, 24 days	Trametinib + Docetaxel Imatinib Epirubicin	Single agent RTK‐inhibitors Everolimus Doxorubicin	Capecitabine Mercaptopurine Thioguanine Trastuzumab Cisplatin Cyclophosphamide
EUP067	F	42	T4N1M1	None	FOLFIRINOX Gemcitabine/Abraxane	14 months, 22 days	Lapatinib + Docetaxel	None	Capecitabine
EUP122	M	64	T4N0M0	Distal pancreatectomy, splenectomy, gastrectomy, vascular resection (CHA), R0	GemCap	34 months, 1 day^a^	None	None	Paclitaxel Methotrexate Tamoxifen
EUP186	M	51	T3N0M0	Distal pancreatectomy, R1	GemCap FOLFIRINOX	47 months, 17 days^a^	FOLFOX FOLFIRINOX MEK inhibitors HSP90 inhibitors PI3K/AKT inhibitors	Singbgble agent RTK Inhibitors Everolimus	Paclitaxel

a Alive as of October 2016.

**Table 3:** Pharmacogenetic biomarkers and mutations identified

Gene	Frequency in these 14 patients	Pathway/action	Potential target therapy	FDA status of targeted therapies	Drug‐biomarker targeted clinical trials	References
*AKT*	5	PI3K/AKT/mTOR	AKT inhibitors. Akt inhibition may modulate platinum‐based therapy resistance	None approved	MK‐2206 in phase II clinical trials, alone, and in combination with platinum‐based chemotherapies. RX‐0201 plus gemcitabine in phase II in advanced PDAC	Cheaib *et al*. (2015), Nitulescu *et al*. (2016)
*APC*	8	Wnt/β‐catenin	WNT inhibitors	FDA‐approved, PDAC off‐label indication. Potentiation of chemotherapy agents by celecoxib and sulindac	Celecoxib with chemotherapy regimines in phase III trials in PDAC	Lesko *et al*. (2014), Lipton *et al*. (2010), Pino *et al*. (2009)
*ATM*	7	Protein kinase. Cell cycle, DNA repair, apoptosis	Associated with either increased or decreased survival when treated with Gemcitabine, depending on variant. Susceptibility to PARP inhibitors. Metformin pathway	Olaparib Metformin	No ATM inhibitors currently in clinical development. Olaparib and rucaparib in phase III trials in PDAC. Phase III trials metformin in combination with chemotherapy	Johnson *et al*. (2016), Mateo *et al*. (2015), Soo *et al*. (2012)
*AURKA*	3	Mediates mitosis	Aurora kinase inhibitors, potentiated by other microtubule‐targeting chemotherapies	None approved	Barasertib and danusertib in phase II clinical trials, SNS‐314 in phase I	Bavetsias and Linardopoulos (2015), Meulenbeld *et al*. (2013), Steeghs *et al*. (2009), VanderPorten *et al*. (2009)
*BRAF*	3	RAF/MEK/ERK signaling, MAPK	BRAF inhibitors, MEK inhibitors	FDA‐approved, PDAC off‐label indication. Vemurafenib and dabrafenib; trametinib. Sorafenib	Sorafenib with erlotinib and sorafenib with gemcitabine plus cisplatin failed phase II trials in advanced PDAC.	Cardin *et al*. (2014), Wilhelm *et al*. (2006)
*BRCA1*	10	DNA repair	Increased susceptability to PARP inhibitors and platinum‐based chemotherapies	FDA‐approved, PDAC off‐label indication for PARPi. Olaparib. Cisplatin och oxaliplatin are FDA‐approved for PDAC, not in monotherapy	Olaparib and rucaparib in phase III trials in PDAC. Veliparib in phase II trials as monotherapy and with gemcitabine and cisplatin	Bendell *et al*. (2015), Kaufman *et al*. (2015), Lowery *et al*. (2011)
*CDA*	1	pyrimidine salvaging, deamination of gemcitabine	Increased gemcitabine toxicity			Hung *et al*. (2012), Nakano *et al*. (2007), Soo *et al*. (2012)
*CDKN2A*	6	Cell cycle	CDK4/6 inhibitor	Palbociclib. FDA‐approved, PDAC off‐label indication	Palbociclib in phase I	Witkiewicz *et al*. (2015a,b)
*DPYD*	9	Inactivation of 5‐FU	Capecitabine, 5‐FU toxicity	FDA‐approved biomarker for adverse drug reaction		Lee *et al*. (2005), Stoehlmacher and Lenz (2003)
*EGFR (ERBB‐1)*	10	MAPK, JNK, PI3K/AKT/mTOR	Predictive role of EGFR intron length and response to anti‐EGFR therapies shown in other cancer types	Afatinib, cetuximab, panitumumab, temsirolimus. Erlotinib is FDA‐approved for use in PDAC, others off‐label in PDAC	Cetuximab in phase III trials no increase in overall survival. Afatinib phase II (togther with MEK inhibitor selumetinib)	Burtness *et al*. (2016), Soo *et al*. (2012)
*ERBB2*	4	MAPK, PKC, JAK/STAT, PI3K/AKT/mTOR, phospolipase Cγ	Her 2/3 inhibitors and antibodies	Afatinib, lapatinib, pertuzumab, (ado‐)trastuzumab emtansine;temsirolimus. Everolimus is FDA‐approved for use in PDAC, others off‐label in PDAC	Trastuzumab with either gemcitabine or capcitabine showed no improval over gemcitabine alone in clinical trial in PDAC	Chou *et al*. (2013), Harder *et al*. (2012), Safran *et al*. (2004)
*FGFR4*	8	Tyrosine kinase receptors	Involved in proliferation and differentiation	FDA‐approved for metastatic melanoma with BRAFV600E mutation	Vemurafenib in phase I trials in PDAC	Hyman *et al*. (2015), Witkiewicz *et al*. (2015a,b)
*KRAS*	12	MAPK	RAF, MEK (*KRAS* V12 mutation and copy number variations are resistant to MEK inhibitors), PI3K or farnesyl transferase inhibitors. Decreased drug sensitivity to erlotinib	Trametinib; tipifarnib, Pantimumab, cetuximab. Selumetinib (orphan drug designation). FDA‐approved, PDAC off‐label indication	Selumetinib similar efficacy to capecitabine advanced PDAC phase II trials. Tipifarnib in phase III showed no improval over gemcitabine in PDAC. R115777 farnesyl transferase inhibitors failed phase II	Bramhall *et al*. (2002), Chiorean and Coveler (2015), Van Cutsem *et al*. (2004)
*MAP3K1*	2	MAPK	MAP3K1 mutation increases sensitivity to platinum‐based chemotherapy and taxanes	None approved	No MAP3K1 modulators currently in clinical development	Hu *et al*. (2014), Li *et al*. (2011)
*MLH1*	8	DNA repair	Decreased sensitivity to 5‐FU and doxorubicin with mismatch repair deficient tumors compared with proficient. Potential susceptibility to platinum‐based chemotherapy, PARP inhibitors	FDA‐approved, PDAC off‐label indication for PARP inhibitor. Olaparib. Cisplatin och oxaliplatin are FDA‐approved for PDAC, not in monotherapy	Olaparib and rucaparib in phase III trials in PDAC. Veliparib in phase II trials as monotherapy and with gemcitabine and cisplatin	Kawakami *et al*. (2015), Nakano *et al*. (2007)
MSH	10	DNA repair	Decreased sensitivity to 5‐FU and doxorubicin with mismatch repair deficient tumors compared with proficient. Potential susceptibility to platinum‐based chemotherapy, PARP inhibitors	FDA‐approved, PDAC off‐label indication for PARPi. Olaparib. Cisplatin och oxaliplatin are FDA‐approved for PDAC, not in monotherapy	Olaparib and rucaparib in phase III trials in PDAC. Veliparib in phase II trials as monotherapy and with gemcitabine and cisplatin	Kawakami *et al*. (2015), Nakano *et al*. (2007)
PALB2	4	DNA repair	PARP inhibitors	FDA‐approved, PDAC off‐label indication for PARPi. Olaparib. Cisplatin och oxaliplatin are FDA‐approved for PDAC, not in monotherapy	Olaparib and rucaparib in phase III trials in PDAC. Veliparib in phase II trials as monotherapy and with gemcitabine and cisplatin	Childs *et al*. (2016), Salo‐Mullen *et al*. (2015), Waddell *et al*. (2015)
*PMS1*	10	DNA repair	Decreased sensitivity to 5‐FU with mismatch repair deficient tumors compared with proficient			Kawakami *et al*. (2015)
*PMS2*	2	DNA repair	Decreased sensitivity to 5‐FU with mismatch repair deficient tumors compared with proficient			Kawakami *et al*. (2015)
*STK11*	2	Regulates polarity, tumor supressor	Metformin pathway	Metformin. FDA‐approved, PDAC off‐label indication	Phase III trials metformin in combination with chemotherapy	Bhaw‐Luximon and Jhurry (2016), Elmaci and Altinoz (2016)

**Figure 1:**
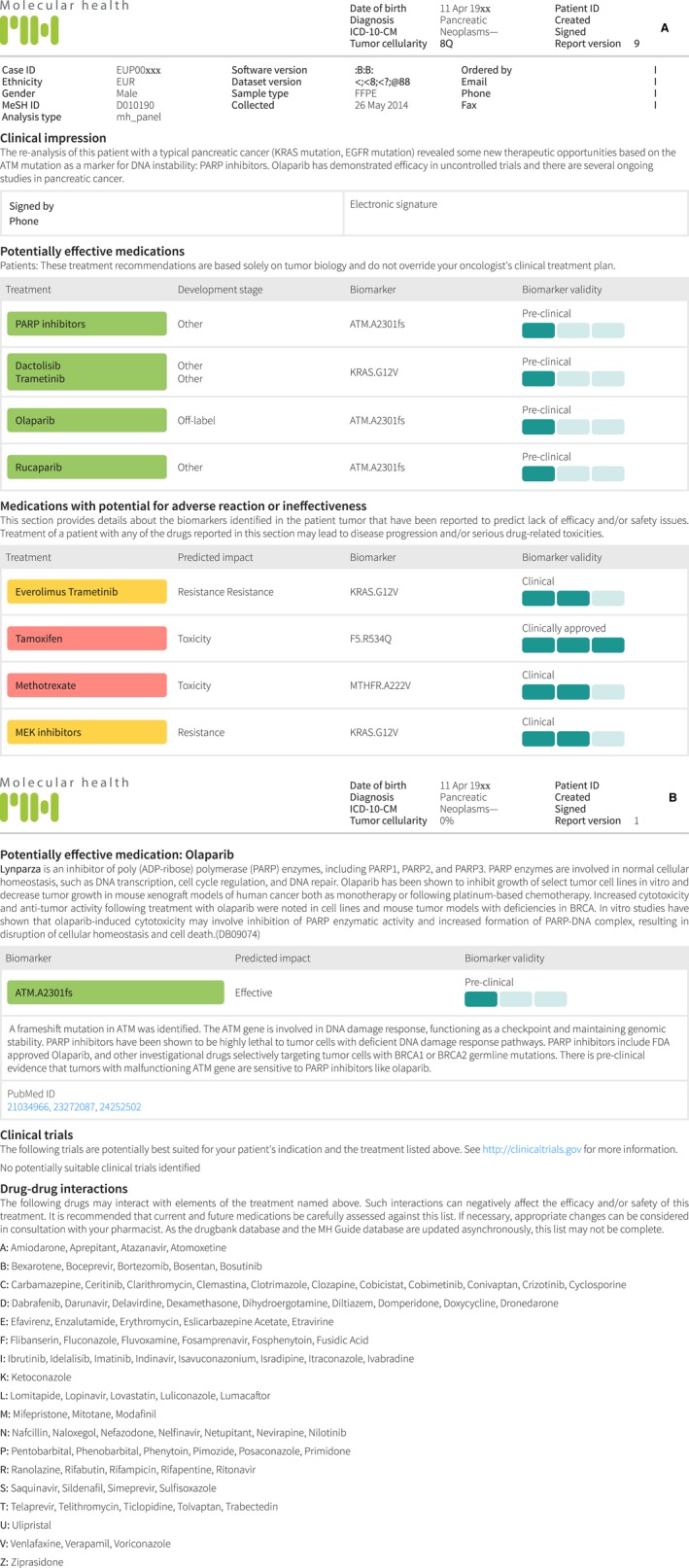
(A) Example of the software‐driven analysis of NGS data resulting in a report that details potentially effective, ineffective or adverse drugs. (B) Example of the more detailed description of a potentially effective drug and drug–drug interactions related to the potentially effective drug.

In all except one patient, drug targets (positive response biomarkers) could be identified with treatmentmap (range 1–8). In all these patients (range 1–4) biomarkers indicating lack of efficacy could be found. In all patients, biomarkers indicating increased toxicity (range 1–5) were found; in six patients, these were FDA‐approved pharmacogenomic biomarkers for toxicity (Fig. 1).

Of the positive pharmacogenomic biomarkers, only everolimus, erlotinib, cisplatin and oxaliplatin are drugs that are FDA‐approved for use in pancreatic cancer. However, several biomarkers indicated off‐label use for approved drugs, as well as suggesting drugs currently in Phase III studies in pancreatic cancer patients. Eighteen biomarkers in 14 patients indicated at least one to three approved drugs in a given patient: trametinib (ABL1.pK266R; *n* = 1) plus docetaxel, imatinib (germline KIT.pM541L), lapatinib, cetuximab, IGF‐1R antibody plus temsirolimus (mTOR inhibitor) as a result of the PTPRD.pR995C, Ewing sarcoma, or an AKT‐inhibitor (MK2206) (KRAS.pG12D; *n* = 13), as well as epirubicin (TP53.pR248Q), paclitaxel (TP53.pR282W) or cisplatin (germline: GSTP1.pI105V). Further drugs in phase III clinical studies that were recommended included PARP inhibitors (olaparib and rucaparib), MEK inhibitors (PD‐0325901 or BAY86‐9766), a PK inhibitor (PF‐05212384), or a combination. Forty‐four biomarkers indicated experimental drugs (pre‐clinical and phase I–III). Because of the *KRAS* mutations, single agent receptor tyrosine kinase (RTK) inhibitors and erlotinib use was predicted to be ineffective in all but one patient. One patient had a mutation in *ERBB4*, and the expression of this gene appears to correlate with non‐metastatic pancreatic cancer and a more favorable outcome (Thybusch‐Bernhardt *et al*., 2001). This mutation was taken as evidence for lapatinib together with docetaxel as a possible treatment option. The combination of lapatinib together with gemcitabine has been studied in pancreatic cancer in a clinical trial that was terminated as a result of ineffectiveness (Safran *et al*., 2011).

Other drugs predicted to be ineffective because of a KRAS.pG12D mutation are everolimus, gefitinib, imatinib (KRAS.pG12D) and adriablastin/doxorubicin (TP53.pR248Q).

Pharmacogenomic biomarkers with evidence for increased systemic toxicity for a series of drugs were detected (Table S3): cisplatin (ERCC2.pD312N, TPMT.pY240C; *n* = 8 patients); capecitabine (DPYD.pC29R, FDA‐approved; *n* = 9); gemcitabine (CDA.pK27Q; *n* = 1); imatinib (ABL1.pK266R; *n* = 1), paclitaxel (CYP2C8.pR139K; *n* = 4); mercaptopurine/thioguanine (TPMT.pA154T, TPMT.pY240C; *n* = 2); trastuzumab (ERBB2.pI655V; *n* = 2); and doxorubicin (CBR3.pC4Y; *n* = 1).

There was a considerable overlap between the drugs suggested by treatmentmap and those recommended by PharmGKB for the patients in the present study. Similarly, there was a strong overlap between the drugs suggested for the entire group by PharmGKB and the drugs that were more probable of demonstrating adverse drug reactions to the patients as individuals based on the treatmentmap data.

## 4 Discussion

For the first time, in an exploratory way, the present study applied NGS with a panel of 620 genes in combination with a novel evidence‐based software tool in the clinical setting of patients with pancreatic cancer. The turnaround time of 2 weeks will enable application for clinical routine use. The quality and quantity of the DNA extracted from FFPE was sufficient to run NGS with good coverage and sufficient reads. Our mutational analysis found the driver mutations known to be frequently altered in pancreatic adenocarcinoma, namely *KRAS*,* TP53*, and *SMAD4* (Witkiewicz *et al*., 2015a,b).

The patients received one of the standard‐of‐care (Seufferlein *et al*., 2012, 2013) chemotherapy regimens for advanced pancreatic cancer (i.e. gemcitabine monotherapy, combination therapy with capecitabine, erlotinib) (Conroy *et al*., 2011a,b) or FOLFIRINOX (5‐fluorouracil, folinic acid, irinotecan and oxaliplatin) (Conroy *et al*., 2011a,b), or gemcitabine in combination with Abraxane (Von Hoff *et al*., 2011), as first‐line therapy, with varying success (Pelzer *et al*., 2011; Zaanan *et al*., 2014). Because this was not an interventional study, no changes in the therapeutic regimen were made based on the NGS/treatmentmap analysis and patients did not receive any of the recommended regimens, with most of them being off‐label uses in pancreatic cancer.

In addition, potential adverse drug reactions yielded several important topical examples where they produced a clear benefit to current treatment recommendations; for example, the DPYD mutations as FDA‐approved biomarkers for toxicity when 5‐fluorouracil (5‐FU) and the oral 5‐FU prodrug capecitabine are used, or cytidine deaminase (CDA) for gemcitabine. Four of the fourteen patients had a predicted toxicity to paclitaxel, which is striking considering that recent studies have shown that the addition of nab‐paclitaxel to standard gemcitabine therapy may provide an additional therapeutic effect in patients with metastatic pancreatic cancer (Von Hoff *et al*., 2013). Also, two of the patients showed genetic susceptibility to an adverse drug event when using FOLFIRINOX, a regimen that has otherwise shown a significant survival advantage when compared to gemcitabine, despite an increased toxicity that perhaps represents the relative commonness of genetic susceptibility to an adverse drug event (Conroy *et al*., 2011a,b).

The results of the present study also clearly demonstrate that precision medicine has several hurdles before it can be expected to be regularly utilized in pancreatic oncology practice (Crane, 2013; Knudsen *et al*., 2015). One such hurdle is the turnover time until the analysis is completed, especially because these patients often have rapid deterioration, as reported from the IMPaCT trial (Chantrill *et al*., 2015). Two patients in the present study did not receive any chemotherapy as a result of rapid deterioration (Chantrill *et al*., 2015). Nevertheless, the turnaround time of 2 weeks appears to be clinically sufficient and feasible, especially in patients undergoing surgery with a postoperative recovery time of around 4 weeks. Another lesson from the IMPaCT trial is to include all drugable targets, and not just concentrate on a few of them. With a median of 30 genetic aberrations in PDAC (Waddell *et al*., 2015) in almost every cellular system and pathway (Jones *et al*., 2008), all mutations should be taken into account, thus requiring an automated analysis to be fast and feasible for clinical use.

In summary, software‐based approaches that include the genetic susceptibility to an adverse drug event and potential ineffectiveness of a number of treatments are becoming increasingly available to clinicians. Precision medicine analyses as reported in the present study may provide opportunities to reduce the costs and time for drug approval by broadening the use of approved drugs to new applications in cancer therapy, or even repurposing noncancer drugs for use in oncology (Lamb *et al*., 2015). In the present study, an evidence‐based analysis of the NGS data of a panel of pharmacogenomic biomarkers revealed potential new therapeutic options for pancreatic cancer therapy. However, most recommended chemotherapeutic agents are currently only used for nonpancreatic cancer malignancies. Additionally, the pharmacogenetic diversity identified in these patients could help explain the lack of treatment response to conventional therapeutic approaches used in pancreatic carcinoma (e.g. cetuximab, imatinib, doxorubicin), as well as the toxicity reported in some.

Taken together, NGS in combination with evidence‐based software analysis of the sequence data is feasible in the clinical setting of pancreatic cancer: unraveling novel treatment options and indicating important biomarkers of increased toxicity.

## Disclaimer

JML serves as contracting physician, in line with current law to use a medicinal class 1 product (software TreatmentMAP). JML is a consultant to Molecular Health GmbH. AP, KS, CH, SB, RB, MS and DJ are employees at Molecular Health GmbH.

## Data Accessibility

Research data pertaining to this article is located at figshare.com: https://dx.doi.org/10.6084/m9.figshare.5311114

## Author contributions

The study was designed by JML and HG. Pathological review of the samples and selection of appropriate tissue was carried out by the pathologists (CFM and CSV) who also confirmed the correct histological diagnosis at that time. DNA extraction was performed by RLH and JL. The panel was designed by JL in collaboration with MS, RB, DBJ and SB. Clinical data were provided by LM, SLH, MJM, MGL and MK. Surgery was performed by MDC. Library preparation and NGS analysis was carried out by JL, VW and LE. The software was designed by AP, MS, SB and DBJ. Data analysis was conducted by KS, AP, CH, RB, MS and SB. Data interpretation was performed by LM, SB and JML. The first draft of the manuscript was written by LM and JML. All authors contributed to various forms of the manuscript and approved the final version submitted for publication. This paper comprises part of PhD thesis of LM.

## Supporting information


**Table S1.** List of included genes.

**Table S2.** Analysis of matched germline and somatic mutations in selected oncogenic cancer syndrome genes (cases with sufficiently good quality sequencing data).

**Table S3.** Germ line variants.
