# Supplementary material for: Bioinformatory‐assisted analysis of next‐generation sequencing data for precision medicine in pancreatic cancer
Source: Mol Oncol. 2017 Aug 8;11(10):1413–29. doi: 10.1002/1878-0261.12108 (PMC5623817; doi:10.1002/1878-0261.12108)
Supplement: Supplementary file 1 — Table S1. List of included genes. [file MOL2-11-1413-s001.docx]

# SUPPLEMENTS

**Table S1:** List of included genes (Excel file)

**Table S2:** Analysis of matched germline and somatic mutations in selected oncogenic cancer syndrome genes (cases with sufficiently good quality sequencing data)

**Table S3:** Germ line variants (Excel file)

**Supplementary Table S2**

| **Gene (hit by variants that are pathogenic (HGMD entries))** | **Frequency in a sub-cohort (9 patients) (Somatic)** | **Frequency a sub-cohort (9 patients) (Germline)** | **Pathway/Action** | **Potential target therapy** | **FDA status of targeted therapies** | **Drug-biomarker targeted clinical trials** | **Ref** |
| --- | --- | --- | --- | --- | --- | --- | --- |
| AKT | 0 | 0 | PI3K/AKT/mTOR | AKT inhibitors. Akt inhibition may modulate platinum-based therapy resistance | None | Phase III PD-0325901 and BAY86-9766. MK-2206 in phase II clinical trials, alone, and in combination with platinum-based chemotherapies. RX-0201 plus gemcitabine in phase II in advanced PDAC. | PMID:26698230 PMCID: PMC4302085 PMID: 20447721 |
| APC | 1 | 0 | Wnt/β-catenin | WNT inhibitors | FDA approved, PDAC off-label indication. Potentiation of chemotherapy agents by celecoxib and sulindac. | Celecoxib with chemotherapy regimines in phase III trials in PDAC. | PMID: 24200292 PMID: 20216081 PMID: 19246950 |
| ATM | 4 | 4 | Protein kinase. Cell cycle, DNA repair, apoptosis | Associated with either increased or decreased survival when treated with Gemcitabine, depending on variant. Susceptibility to PARP inhibitors. Metformin pathway | Olaparib. Metformin. FDA approved, PDAC off-label indication. | No ATM inhibitors currently in clinical development. Olaparib and rucaparib in phase III trials in PDAC. Phase III trials metformin in combination with chemotherapy. | PMCID: PMC3795429 PMID: 26510020 |
| AURKA | 4 | 6 | mediate mitosis | Aurora kinase inhibitors, potentiated by other microtubule-targeting chemotherapies | None | Barasertib and danusertib in phase II clinical trials, SNS-314 in phase I | PMID: 19372566 PMID: 19770380 PMC4685048 PMID: 22928785 |
| BRAF | 1 | 0 | RAF/MEK/ERK signaling, MAPK | BRAF inhibitors, MEK inhibitors | FDA approved, PDAC off-label indication. Vemurafenib and dabrafenib; trametinib. Sorafenib. | Sorafenib with erlotinib and sorafenib with gemcitabine plus cisplatin failed phase II trials in advanced PDAC. | PMID: 17016424 PMID: 24574334 PMID: 20803052 |
| BRCA1 | 3 | 6 | DNA repair | Increased susceptability to PARP inhibitors and platinum-based chemotherapies. | Olaparib. FDA approved, PDAC off-label indication for PARPi. Cisplatin and oxaliplatin are FDA approved for PDAC, not in monotherapy. | Olaparib and rucaparib in phase III trials in PDAC. Veliparib in phase II trials as monotherapy and with gemcitabine and cisplatin. | PMID: 25573533 PMID: 25366685 PMID: 21934105 |
| CDA | 0 | 0 | pyrimidine salvaging, deamination of gemcitabine | Increased gemcitabine toxicity. |  |  | PMCID: PMC3795429 PMID:17224927 PMID: 22425961 |
| CDKN2A | 0 | 0 | Cell cycle | CDK4/6 inhibitor | Palbociclib. FDA approved, PDAC off-label indication. | Palbociclib in phase I. | PMID: 26158861 |
| DPYD | 4 | 8 | inactivation of 5-FU | Capecitabine, 5-FU toxicity | FDA approved biomarker for adverse drug reaction. |  | PMID: 12749725 PMID: 15709212 |
| EGFR (ERBB-1) | 4 | 4 | MAPK, JNK, PI3K/AKT/mTOR | predictive role of EGFR intron length and response to anti-EGFR therapies shown in other cancer types | Afatinib, cetuximab, panitumumab, temsirolimus. Erlotinib is FDA approved for use in PDAC, others off-label in PDAC. | Cetuximab in phase III trials no increase in overall survival. Afatinib phase II (togther with MEK inhibitor selumetinib). | PMCID: PMC3795429 PMCID: PMC4177955 |
| ERBB2 | 2 | 2 | MAPK, PKC, JAK/STAT, PI3K/AKT/mTOR, phospolipase Cγ | Her 2/3 inhibitors and antibodies | Afatinib, lapatinib, pertuzumab, (ado-)trastuzumab emtansine;temsirolimus. Everolimus is FDA approved for use in PDAC, others off-label in PDAC. | Trastuzumab with gemcitabine showed no improval over gemcitabine alone in clinical trial in PDAC | PMID: 15581051 |
| KRAS | 8 | 0 | MAPK | RAF, MEK (*KRAS* V12 mutation and copy number variations are resistant to MEK inhibitors), PI3K or farnesyl transferase inhibitors. Decreased drug sensitivity to erlotinib. | Trametinib; tipifarnib, Pantimumab, cetuximab. Selumetinib (orphan drug designation). FDA approved, PDAC off-label indication. | Selumetinib similar efficacy to capecitabine advanced PDAC phase II trials. Tipifarnib in phase III showed no improval over gemcitabine in PDAC. R115777 farnesyl transferase inhibitors failed phase II. | PMID 15084616 PMID 12107836 PMC4500614 PMID: 21594619 |
| MAP3K1 | 0 | 0 | MAPK | MAP3K1 mutation increases sensitivity to platinum-based chemotherapy and taxanes | None | No MAP3K1 modulators currently in clinical development | PMID: 21636554 PMID: 24253898 |
| MLH1 | 6 | 4 | DNA repair | decreased sensitivity to 5-FU and doxorubicin with mismatch repair deficient tumors compared with proficient. Potential susceptibility to platinum-based chemotherapy, PARP inhibitors | FDA approved, PDAC off-label indication for PARP inhibitor. Olaparib. Cisplatin and oxaliplatin are FDA approved for PDAC, not in monotherapy. | Olaparib and rucaparib in phase III trials in PDAC. Veliparib in phase II trials as monotherapy and with gemcitabine and cisplatin. | PMID: 17224927 PMID: 20823149 |
| MSH | 1 | 5 | DNA repair | decreased sensitivity to 5-FU and doxorubicin with mismatch repair deficient tumors compared with proficient. Potential susceptibility to platinum-based chemotherapy, PARP inhibitors | FDA approved, PDAC off-label indication for PARPi. Olaparib. Cisplatin och oxaliplatin are FDA approved for PDAC, not in monotherapy. | Olaparib and rucaparib in phase III trials in PDAC. Veliparib in phase II trials as monotherapy and with gemcitabine and cisplatin. | PMID: 20823149 |
| PALB2 | 2 | 3 | DNA repair | PARP inhibitors | FDA approved, PDAC off-label indication for PARPi. Olaparib. Cisplatin and oxaliplatin are FDA approved for PDAC, not in monotherapy. | Olaparib and rucaparib in phase III trials in PDAC. Veliparib in phase II trials as monotherapy and with gemcitabine and cisplatin. | PMID: 27197284 PMID: 26440929 PMID: 25719666 |
| *PMS1* | 0 | 0 | DNA repair | decreased sensitivity to 5-FU with mismatch repair deficient tumors compared with proficient |  |  | PMID: 26031544 |
| PMS2 | 2 | 4 | DNA repair | decreased sensitivity to 5-FU with mismatch repair deficient tumors compared with proficient |  |  | PMID: 26031544 |
| *STK11* | 1 | 0 | Regulates polarity, tumor supressor | metformin pathway | Metformin. FDA approved, PDAC off-label indication. | Phase III trials metformin in combination with chemotherapy. | PMID: 27377891 PMID: 27255657 |
| PRSS1 | 0 | 1 | pancreatic enzyme activation | none | n/a | n/a | PMID: 8841182 |

## Supplementary File: Experimental procedures

### Details on the NGS validation

The technical and clinical (diagnostic) accuracy of a specific implementation of NGS measurements of genomic DNA from patient (tumor) samples (including FFPE material) using MH´s EngineusPANEL 600+ in combination with the data analysis by TreatmentMAP engine (TME) was determined. Validation (or benchmarking) results were used to allow a performance description adequate for the declaration as in vitro diagnostic medical device. We have shown that the medical device (MH´s EngineusPANEL 600+) fulfills the “Essential Requirements” as stated in the Directive 98/79/EC about IVD medical devices.

The standard MH validation comprises measurements of the following materials:

A. HorizonDx HD200, "NGS Quantitative Multiplex Reference Standard”, available as FFPE slides. It has 35 known variants with known VAF’s and independently verified by droplet digital PCR.

B. HG001 and HG002 from the Genome in a Bottle (GiaB). DNA for both is available from Coriell; for the first as HapMap NA12878, for the second as NA24385. Analysis was done of pure samples as well as of mixtures (1:2; 1:4; 1:8; 1:16).

C. Clinical samples. All of them are independently partially verified by measurement according to the OncoCarta Panel v1.0.

A total of 40 measurements are included in this validation:

- 4 replicate measurements of the HorizonDX HD200 material (FFPE);
- 9 measurements of 6 samples created from reference material from Genome in a Bottle (DNA from cell lines); and
- 27 measurements of 19 clinical tumor samples (FFPE material).

All measurements passed the quality checks and showed normal values for a whole array of quality parameters.

For each of the samples, we have partial knowledge on SNVs that should be present and on SNVs that should be absent in the sample; this is what we call ground truth. This knowledge is derived in different ways for the three types of samples:

- For the GiaB materials (HG001 and HG002), ground truth is determined by a thorough analysis of the datasets that are provided by the GiaB Consortium on <https://sites.stanford.edu/abms/content/giab-reference-materials-and-data/>. The details of this analysis are provided in a separate document.
- For the GiaB mixtures, ground truth is computed as the linear combination of the ground truths of the individual materials according to the mixture ratios.
- For the clinical samples, we ordered OncoCarta analyses of the FFPE material from an independent provider.
- For HorizonDX, we use the information provided by the manufacturer (Horizon).

Accuracy:

The IVD provides SNV calls that are accurate: most reported SNVs are correct (high PPV), and most of the SNVs present in the material are reported (high sensitivity).

- At the desired minimum coverage level (500x avg diagnostic coverage), the accuracy of SNV calling was found to be as shown in the following table.

| **Material** | **PPV_est** | **PPV_CI** | **Sens_est** | **Sens_CI** |
| --- | --- | --- | --- | --- |
| HorizonDX | 100,00% | 82.35%--100.00% | 100,00% | 82.35%--100.00% |
| GiaB | 98.03% | 97.78%--98.26% | 98,40% | 98.17%--98.61% |
| Clinical | (100.00%) | (75.29%--100,00%) | (92.86%) | (66.13%--99.82%) |

Notes:

- Note 1: The values for the clinical samples are put in parentheses, as they are based on the presumed result of pending verification measurements. See Section 6.6 for details. These values are in better agreement with observations on the other two types of material than presuming that all differences are due to mistakes by the MH IVD and that OncoCarta would be 100% free of errors.
- Note 2: The intended use of the IVD is the analysis of clinical samples, which are typically prepared as FFPE blocks. Hence the accuracy estimates from such clinical material are most relevant and applicable for the intended use. However, these estimates necessarily are based on low numbers of comparisons to ground truth. They therefore come with a large degree of uncertainty, which is manifest in wide confidence intervals. Only the GiaB reference material allows to determine narrow confidence intervals, ie precise estimates of SNV detection accuracy. While the accuracy on the high quality GiaB material is expected to be higher than the accuracy on FFPE material, it does represent the entire processing chain starting from the extracted DNA and it quantifies the accumulated errors that can occur in all the steps from DNA to called SNVs. We therefore believe that it provides a good indication of where the true accuracy on clinical FFPE material lies within its wide confidence interval. Given the data gained from this validation, this would imply clinical PPV and sensitivity relatively close to 100%.
- We have also shown that very high levels of accuracy are achieved for high allele frequency SNVs and are maintained down to about 10% AF.
- As investigated for the GiaB samples, sequencing to a reduced average diagnostic coverage 200x still supports accurate SNV calling: we found values of PPV and sensitivities to be well above 90% except for low allele frequencies. This suggests that the IVD can also provide reliable results for low quality sample material from which only low diagnostic coverage sequence data can be produced.
- Finally, the allele frequency estimates computed by TME are in reasonable agreement with ground truth knowledge on the true AFs.
